# Learning: One definition to rule them all?

**DOI:** 10.1038/s41539-026-00441-7

**Published:** 2026-07-15

**Authors:** Martin Lövdén, Isabelle Hansson, Toms Voits

**Affiliations:** Department of Psychology, University of Gothenburg, Gothenburg, Sweden

**Keywords:** Language and linguistics, Language and linguistics, Psychology, Psychology

## Abstract

Learning is central across many disciplines, yet its definition remains fragmented, hindering theoretical progress and cross-disciplinary communication. We review debates on the definition of learning and propose a unifying, umbrella, definition: learning is a system’s processing of information from its environment, resulting in a change of system properties that alter its behavioral potential and reactions to this information. This formulation supports interdisciplinary dialogue while allowing discipline-specific operationalizations.

Learning is a central concept in many scientific disciplines, including psychology, educational science, neuroscience, biology, sociology, evolutionary theory, computer science, and the interdisciplinary endeavor of learning sciences. This concept is at the heart of the most fundamental research problems, such as dissolving the nature-nurture dichotomy in ontogenetic development, explaining cultural evolution, and improving human and artificial intelligence. It is therefore surprising that researchers’ understanding and definitions of “learning” vary considerably^1–7^. For example, Barron and colleagues^2^ list more than 50 different definitions of learning that can be found in psychology, neuroscience, behavioral ecology, and computer science. Several handbooks of learning have given up on the task of defining the term^5,7^. The multidisciplinary journal “npj *Science of Learning”* does not offer a definition either, despite learning and adjacent topics being at the very core of its aims and scope.

It can be argued that people share at least an implicit concept of learning, given that both researchers and laypersons are generally able to communicate using the term. In everyday usage, learning is commonly understood in a way that aligns with what learning scientists describe as an instructionist perspective; that is, learning is associated with the absorption and retention of information. This is reflected in the convergence of many textbook and dictionary definitions. For example, Oxford Languages defines learning as “the acquisition of knowledge or skills through study, experience, or being taught.” Similarly, the Wikipedia entry on learning, citing Gross’s Psychology: The Science of Mind and Behaviour, defines learning as “the process of acquiring new understanding, knowledge, behaviors, skills, values, attitudes, and preferences.”. However, these definitions are deceptively straightforward, as they rely on concepts, such as knowledge, skill, and understanding, that are themselves difficult to define precisely. Moreover, folk understandings of learning are not uniform. Säljö^8^, for example, demonstrated substantial variation in responses to the question “What do you actually mean by learning?” The nature of these responses was related to educational background, with higher levels of education associated with greater reflection and abstraction concerning the meaning of learning. Thus, even everyday conceptions of learning appear to vary considerably across individuals.

The polysemy of the learning term comes with serious costs. Variability in the meaning of terms hinders within- and cross-disciplinary communication and knowledge accumulation^2,9^. Substantial scientific progress is also forfeited if the task of defining the concept is treated lightly. Strict definitions of concepts are often critical for theory development. For the learning concept, the variability in understanding carries substantial ontological importance^3^. The research community must therefore take the task of finding consensus on the meaning of the learning term seriously. The timing is right. Although the definition has been discussed for a very long time^10–14^, several papers published during the last ten years have laid a good foundation for coming to a consensus^2,3,9^.

Faced with our confusion regarding how to define the concept of learning in a useful way, we decided to take a step back and first establish a set of criteria for what constitutes a good cross-disciplinary concept such as learning. Equipped with these desiderata, we then review the existing literature on the definition of learning, asking whether the many existing definitions could be consolidated into a unifying and useful concept applicable across scientific disciplines, organisms, humans and machines, as well as individuals and groups. The need for such a cross-disciplinary concept is particularly evident in an era in which artificial systems exhibit increasingly complex adaptive behaviors. Our goal is to stimulate interdisciplinary discussion and encourage researchers to explore the utility of our proposed “umbrella” definition of learning within their own fields of research.

## What makes a useful concept and a good definition?

Concepts are building blocks of human thought and of scientific theories. Many traditions maintain that, for scientific theories to be complete, concepts need operational definitions that prescribe how the concepts are manifested in reality^15,16^. To guide the development of operational definitions, good concepts that are well defined by verbal statements are helpful. This begs the question of what constitutes a good scientific concept and a good definition of a concept.

## What makes a concept useful?

Achieving cross-disciplinary consensus and applicability are valuable overarching goals for a concept such as learning that is used in many disciplines. Skau and colleagues^17^ listed a few desiderata for achieving this goal. First of all, it is important that the concept is discipline-neutral. For the concept of learning, we argue that it should therefore be applicable to different systems, such as cellular networks, organisms, groups, and machines. Skau and colleagues^17^ also considered the ease of operationally defining concepts. Such a desideratum may however restrain theoretical development. In the history of science, there are many examples (e.g., “atom” and “gene”) of concepts that have been immensely valuable for scientific progress, although they were initially hard to operationally define. The ease of operationally defining concepts is therefore desirable, but not essential, especially in a cross-disciplinary context where operational definitions are likely to differ across disciplines. We argue that a broad concept is meaningful for facilitating cross-disciplinary consensus. This umbrella concept may be narrowed down in different disciplines and subfields^2^, such that they are congruent with the umbrella concept but distinguish between different forms of it. Further, to ease communication, cross-disciplinary scientific concepts should not conflict with prevailing folk understanding. Though scientific progress sometimes has been facilitated by letting go of the everyday meaning of concepts^9^, such concepts and their definitions often become too obscure to facilitate cross-disciplinary communication. This holds especially for agreeing on the phenomenon we must explain (i.e., the explanandum), although explanations for the phenomenon (i.e., explanans) almost always require more nerdy thoughts^18^. For cross-disciplinary concepts, we agree with Skau and colleagues^17^ that the definition should preferably describe an explanandum rather than be proposals for explanans. Indeed, it appears to be easier to achieve consensus regarding the nature of a phenomenon than it is to agree on the explanations for why it occurs^18^. It is further important that the definition captures the essential nature of the phenomenon^19^, assuming that there is a true core of learning. A minimum requirement for this is that it reflects the current state of knowledge.

## What makes a concept well defined?

The above discussion brings us to also nailing down desiderata for a good definition (i.e., the statement of the meaning) of the learning term. The definition should be precise, such that it is applicable to everything to which the concept applies, but to nothing else. For learning, the definition should, for example, probably be distinct enough such that it does not encompass phenomena such as maturation and senescence. Circularity should clearly be avoided. Obscure definitions are also not desirable. That is, the meaning of things should be defined by words that are commonly understood and have good definitions, parts of the definition itself may need a definition. It is preferable that the definition is positive. A negative definition, stating what a concept is not, will need additional definitions (and a long and almost certainly incomplete list of what the concept is not about).

## Discussions surrounding the definition of learning

There is reasonable consensus among both lay persons and researchers that an essential property of learning is that it is a process, and neither a capacity (i.e., ability) nor a result (i.e., product) of a process^20–22^. A process can be defined as *systematic series of events or set of events that interact and develop over time*^3^. A process results in a product of a quality or quantity that did not exist before; that is, in a change. Some systems do not have the capacity for this process, but others have the ability to learn. In systems that have the capacity, the process can differ in efficiency; that is, how good the product of a process is given the amount of input/energy used^23^. Though there is reasonable agreement on these statements among researchers from different disciplines, there is disagreement on whether the definition of learning requires only reference to a specific process or whether the input and output of the process must be defined, and, if so, how the input and output should be defined.

## Defining the output – functional and structural perspectives on learning

A crucial divergence in the existing definitions of learning relates to whether the product of a system’s learning process is a functional or structural change^3,11^. A *functional* change refers to a change in the behavior of a system (the product is a behavior that has been learned, a learned behavior). A *structural* change refers only to the internal process of changes in the features of the system (i.e., the product is a state of the system that has been learned, a memory). Some of the functional definitions also include structural components, or at least an explicit or implicit reference to some kind of internal process accompanying behavioral change^3^.

Classic learning theories can be broadly situated along this distinction. Behaviorist approaches (e.g^12^.,) define learning in functional terms as changes in behavior, whereas Piaget’s^24^ theory emphasizes structural change in the form of transformations of cognitive schemas. Social learning theory^25^ foregrounds functional change while invoking internal mediating processes. Sociocultural and activity-theoretical approaches^26,27^ integrate both perspectives by conceptualizing learning as changes in socially mediated activity that become internalized or reorganized within systems. Situated learning theories^28^ further shift the emphasis toward functional changes in participation within social practices, with structural change residing in evolving relations within communities. Conceptual change approaches similarly emphasize structural transformation, characterizing learning as qualitative reorganization of knowledge systems rather than incremental behavioral adjustment^29,30^.

Functional definitions have several advantages. They describe how learning is manifested (i.e., an explanandum) and no knowledge of the internal features of the system is needed for verifying that learning has occurred^4,9,12^. Most importantly, multiple realizability of internal structures and mechanisms (cellular networks, computer algorithms, evolutions, groups) that produce the same behavior is not an issue for functional definitions^9^. Learning may, however, occur without behavioral change, which presents a significant problem for functional definitions (the “behavioral silence” problem). For example, the system may have learned something (i.e., stored a memory) without demonstrating any change in its behavior^3,5,21^.

If learning can occur without behavioral change, then a functional definition does not sufficiently capture the full scope of learning. Instead, it is reduced to a proxy of an operational definition of learning^3^. That is, behavior is studied to make inferences about the internal process of the system (i.e., changes in the internal state of the system, such as changes in connection strength; Burgos, 2018) that we cannot always study directly. In this sense, functional definitions therefore do not cover the true processing essence of learning^31^ – learning is an internal process, which is merely evidenced by behavioral change^3,32^. A functional definition would also alienate most lay persons and researchers (not least those in cognitive and educational science) who consider learning a process for storing information (i.e., memory/knowledge). In fact, even most proponents of functional definitions would probably agree that a functional change must always have a structural correlate. Purely functional definitions, such as the one by De Houwer & Hughes (2023), who define learning as “changes in the way a system behaves toward a stimulus that result from regularities in the environment of that system”, thus seem at odds with the common understanding of learning. It is more an operational definition or a definition of “learned behavior” (i.e., the product of a process).

It thus seems prudent (and necessary) to acknowledge the structural process when defining learning. This does not mean that we must restrict learning to specific structural changes, because that would decrease cross-disciplinary applicability. A pure structural definition (e.g., changes in connection weights in a neural network) does indeed run the risk of becoming too narrow to work well as a cross-disciplinary concept. Structural definitions tend to be, more often than functional definitions, formulated as explanations rather than descriptions of learning. Alternatively, they tend to be too general, running a higher risk than functional definitions of becoming vague. For example, structural definitions often include reference to concepts describing internal structures, such as memory and knowledge, that are difficult to define. A prominent example of such a definition is Barron’s^2^ definition of learning as “a structured updating of system properties based on processing of new information”.

We think a solution for the definition of learning is to combine structural and functional definitions. In such a combined definition, the structural aspect is acknowledged, but not specified, as the true essence of learning, while the functional definition helps to cover more disciplines and guide operational definitions. Definitions like this exist, such as the definition by Alexander and coleagues^1^: “Learning is a multidimensional process that results in a relatively enduring change in a person or persons, and consequently how that person or persons will perceive the world and reciprocally respond to its affordances physically, psychologically, and socially”, but they are often limited in scope (e.g., restricted to humans). Moreover, we still need a solution that can potentially save the functional definition from the behavioral-silence problem. One such solution is to specify that learning results in a change in the range of possible behavior^33–35^. This solution introduces an explicit latent aspect to the definition, leaving the operationalization of the potential behavior to a measurement problem. While this may lead to vagueness^21^, a theoretical definition is about a latent construct, and should be separated from the operational definition. Also, operational definitions of the range of behavior are not in any way impossible to develop (see, for example, “reaction norms”; e.g.^36^). Thus, to summarize so far, learning is a process in a system that results in a change of system properties and its range of possible behavior.

## Defining the input – What makes an output to qualify as being learned?

Another substantial discussion in the literature hovers around what kind of input to the process makes the outcome to qualify as being learned. In researchers’ and lay persons’ worlds of concepts, systems can change their behavioral potential without learning. For example, an organism’s range of performance may change (for better or worse) due to injury, hormonal influence, maturation, and/or senescence. Changes induced by these causes are typically not considered to be learned, with maturation, senescence, and learning generally treated as distinct processes. Past functional definitions of learning have therefore sometimes included negative qualifiers, listing processes not included in the concept. This is not a desirable way forward. The solution is to include a positive qualifier, restricting learning to a process that is based on specific input. Definitions taking this route often restrict learning to a process that is based on extracting information from experience. Such a definition has the advantage of being system-general and applicable not only to organisms but also machines^3^. However, experience is not a trivial concept to define, and, when defined, many representations in a system are often assumed. Information somehow must reach the system that is learning, but the word “experience” raises connotations of awareness (few would accept that computers are experiencing), and learning is possible without awareness. Alternatives, such as activation in the system or sensed environments, are also not easily defined. What constitutes the environment of a system may be quite easy to define in theory, but is rarely easily operationally defined. If we define a system as a group of interacting or interrelated elements that work according to a set of principles to form a unified whole, then an organism (e.g., a human) or a machine (e.g., a robot) may be one supersystem (or, group) that consists of several subsystems (or, group members; e.g., organs, neural networks, and genes), implying that the supersystem could learn from internal processes (i.e., information transmitted from one subsystem may be the environmental input for another subsystem). However, a critique of this conclusion can be that the ultimate cause of this learning is environmental (i.e., external to the supersystem). In this sense, the “learning by thinking”^37^, or “representational redescription”^38^, is simply a deeper description of a specific form of learning process that gets its original food for the process from once learned information from the environment (i.e., stored information).

A potential solution to the problems of defining experience and environment of a system that works across disciplines is to anchor the definition in information theory, simply specifying that learning relies on the processing of information^2^. However, this would make the concept very broad and the definition imprecise and inconsistent with folk understanding. Many processes typically not counted as learning (e.g., gene expression in maturation, hormonal influences) surely require some kind of information processing at some level (although it would exclude some effects, such as, for example, injuries). It thus seems necessary to restrict learning to at least being based on the processing of information from the environment of a system. A system with a capacity for learning (e.g., nervous system, hidden layers of neural networks) not only needs a learning process (e.g., updating of connection strength) but also the ability to detect information from its environment (e.g., task for the computer program or sensed information from the natural environment). This necessary condition would exclude genetically programmed maturation while allowing for learning in gene-environment interactions during maturation, such as the development of sensory systems programmed to expect information through the senses during maturation (i.e., experience-expectant plasticity). Learning is therefore not restricted to the development of specific skills (i.e., experience-dependent plasticity^39^;). Thus, so far, learning is a system’s processing of information from its environment. This process results in a change of system properties and its range of possible behavior.

A remaining problem is that most people probably think that learning changes the way a system reacts to information. For example, a human who encounters the statement that Paris is the capital of France will react differently to this the first time as compared to the second time the statement is encountered, if the information has been learned the first time. In a similar vein, direct hormonal influences on a system are typically not considered to be an example of learning, but if the system reacts in a different way the next time the system gets the same hormonal influence, then most people would consider this a learned response. Common solutions to this problem in the past have been to define learning as changes in behavior towards a stimulus^9^, but these solutions place the definition firmly in the functional corner. When placed in the context of information theory and structural definitions, solutions have been to specify that the information must be new^2^, but what is novel information and what is not is easy to define and surely, a system can continue to learn from repeated presentation of the same information. It therefore seems more appropriate to describe learning in terms of changes in how the system reacts to the information it has processed. This restriction does not mean that learning cannot also materialize in other changes, but just that it has to react differently to repeated information. The alteration in how the system reacts to information from its environment is the final piece of the definition of learning that we consider.

## Toward a unifying definition of learning

Based on our review of discussions surrounding the concept of learning, we propose a unifying definition of learning as *a system’s processing of information from its environment, resulting in a change of system properties that alter its behavioral potential and reactions to this information*. It is designed to capture the essential nature of learning (as a process) and the core components necessary for learning to occur (specification of input and output components). It is intended to serve as an umbrella definition that can be applied across different scientific disciplines, while allowing for theoretical diversity in specifying the underlying mechanisms. In this section, we further evaluate the proposed definition in relation to our desiderata for a good cross-disciplinary concept that is well defined.

The definition relies on the concept of information, but we do not think this excludes any discipline that studies learning. It is applicable to both organisms and machines, and to groups, if we simply define groups as systems (the group) consisting of interacting subsystems (the group members^40^;). It also excludes processes like maturation and senescence, to the extent that these are not based on the processing of information from the environment. It is thus a broad definition, but not one that is devoid of meaning and distinctiveness. It may serve as an umbrella concept that can be further specified in different disciplines without deviating from the core components in the umbrella definition. It has two terms, *process* and *system*, that we have defined above, and there is reasonable agreement on the meaning of these terms, but there are additionally two terms that may need discipline-specific definitions to reach acceptable precision: *behavior* and *information*. Information is a term that opens a can of worms, and defining behavior is an intimidating task^3^, although there are good suggestions^9,41^. On the other hand, information and behavior are terms that lay people have a relatively unified and common understanding of. The same goes for process and system – they are terms that need definition, but not in the context of functioning as guiding umbrella concept. The terms therefore function well in a cross-disciplinary context, although applications may need further specification.

The definition combines references to structure, which is consistent with folk understanding of learning in the sense of storing knowledge, with the advantage of a functional component that guides operational definitions. It is not obscure in the sense that it cannot be understood, although it excludes terms that lay persons would expect in relation to learning, such as memory and knowledge. On the other hand, these terms are often poorly defined internal states and poorly applicable across systems, and they are allowed as explanations of the change in the range of possible behavior in the definition. There is no circularity, and it does not require a list of processes to be excluded. The definition offered is what we as researchers must explain. It captures the essence of many definitions in the literature, including learning as the processing of information from the environment and the aspect of behavioral change^2^, but avoid references to specific mechanisms of change that sometimes are mentioned. We thus think the definition strikes a good balance between our desiderata for a good cross-disciplinary concept that is well defined.

## Concluding remarks

In this article, we asked the question whether it is possible to develop a unifying definition of learning that can be applied across different scientific disciplines. We believe that it is and argue that cross-disciplinary agreement on the essence of learning constitutes an important step for facilitating interdisciplinary dialogue and advancement. The proposed definition—*a system’s processing of information from its environment, resulting in a change of system properties that alter its behavioral potential and reactions to this information*—is designed to serve as an umbrella definition that bridges gaps between traditional behaviorist and modern computational perspectives, making it more applicable across diverse systems, including biological organisms, artificial networks, and social groups. We thereby hope to contribute to cross-disciplinary discussion and inspire scholars to explore its applicability within their own field of research.

## Data Availability

No datasets were generated or analysed during the current study.
